# The Long-Term Impact of Family Experiences in Adolescence on Mother–Child Relationships in Early Adulthood

**DOI:** 10.1177/0192513X251330609

**Published:** 2025-04-11

**Authors:** Fred Berger, Romain Jammal-Abboud

**Affiliations:** 1 Department of Education, 27255 University of Innsbruck, Innsbruck, Austria; 2 61315 Zefat Academic College, Zefat, Israel; 3 26745 University of Tel Aviv, Tel Aviv, Israel

**Keywords:** family experiences in adolescence, parental divorce and separation, mother–adult child relationship, long-term effects, prospective longitudinal study, structural equation modeling

## Abstract

In this study, we examine the long-term effects of family experiences during mid-adolescence, including parental divorce and separation, on mother–child solidarity in early adulthood. The study combines a longitudinal analysis of stability and change in family relationships from mid-adolescence to early adulthood with a cross-sectional examination of the mechanisms of intergenerational solidarity in early adulthood. The data come from the German LifE-Study, which spans 20 years (from 1982 to 2002) and includes information on 1179 mother–child relationships. Findings from structural equation models reveal that emotional closeness, contact frequency, and instrumental support provided by adult children to mothers are significantly influenced—both directly and indirectly—by family experiences in adolescence. Additionally, compared to mother–adult daughter relationships, mother–adult son relationships were found to be less involved and more reliant on current contact frequency. However, for most participants, the mother–adult child relationship was found to be close and supportive, constituting a long-lasting bond.

## Introduction

It is now well supported by scientific evidence that parenting and parent–child relationships during childhood and adolescence have a significant impact on children’s and adolescents’ development (Sanders & Morawska, 2018). However, not studied as extensively in the research is the question of the long-term influences of the early history of the parent–child relationship on the later intergenerational relation. The expectation is that close and supportive parent–child relationships during childhood and adolescence should be linked to high levels of emotional closeness and, when residential proximity is controlled, to frequent contact, shared activities, and mutual support in later intergenerational relations (Antonucci et al., 2013). To date, only few prospective studies allowed the investigation of parent–child relations over a longer time period (Bengtson et al., 2002a; Collins et al., 2012; Pillemer & McCartney, 2013). Most previous studies were based upon retrospective data or examined only a few years of development. In contrast, this study draws on data from the German prospective longitudinal LifE-Study, which spans 20 years from 1982 to 2002 (Fend et al., 2009).

In this study, we propose and empirically test a model that aims (1) to uncover the long-term impact of family experiences in mid-adolescence—including parental divorce and separation—on later mother–child relationships and (2) to examine the associations among emotional closeness, frequency of contact, and instrumental support provided by adult children to their mothers in early adulthood. The central question is: to what extent, and in what ways, do family interactions, as well as parental divorce and separation during childhood or adolescence, influence these different dimensions of mother–child relationships in early adulthood. Special attention is given to differences between mother–daughter and mother–son relationships.

The analyses focus on mother–child relationships from mid-adolescence to early adulthood because the transition from adolescence to adulthood is considered a sensitive development period for parent–child relationships (Collins et al., 2012). During adolescence, it is critical that increasing symmetry and a new balance between relatedness and autonomy can be established in the parent–child relationship. The relationship must evolve from one characterized by childlike dependency to one between adults. It must undergo transformation to remain significant to the younger generation (Branje, 2018). Furthermore, mothers have been found to play a special role in the fabric of family relationships (Cooney & Dykstra, 2012). According to many studies from Western countries, mothers invest more in maintaining family relationships than fathers do (Carr & Utz, 2020; Silverstein & Giarrusso, 2010). As individuals age, the same pattern holds for daughters compared to sons (Carr & Utz, 2020; Cooney & Dykstra, 2012). In women’s intergenerational relationships, previous studies have found strong continuity in emotional closeness and contact, as well as a high willingness to provide mutual support in adulthood (Cooney & Dykstra, 2012; Fingerman et al., 2020; Swartz, 2009).

## Theory

Research on the impact of early family experiences on later parent–child relationships is very much inspired by social learning theory and attachment theory. These theories provide an understanding for the association of early patterns of parent–child interactions with later intergenerational relations. Social learning theory holds that core patterns of interaction (e.g., communication and supporting behavior) are learned through observation, imitation, and positive social reinforcement and established in family life when children are young. These patterns persist over time and are invoked in new situations such as when parents are in need of support in later life (Bandura, 1986; Schönpflug & Bilz, 2009). Attachment theory suggests that experiences of care and parenting within the parent–child relationship in childhood and adolescence are internalized and stored as internal blueprints of both self and relationships with others (Bowlby, 1978; Merz & Jak, 2013). Although other significant relationships develop throughout life, these blueprints endure and pre-determine subsequent relations with family members and individuals outside of the core family unit (Feeney & Woodhouse, 2016). The unique relationship between parent and child, developed in childhood and adolescence, is believed to motivate continued intergenerational closeness, and later contact and support in adulthood (Shaver et al., 2016).

Most theoretical arguments on the long-term effects of parental divorce and separation have focused on the role of the father. There is less attention given to how the mother’s ties to the children are affected by divorce or separation. A central argument for why both divorced or separated fathers and mothers are negatively affected relates to opportunities. Adult children with married parents can visit both parents together or provide support to them simultaneously. However, for children of divorced or separated parents, such economies of scale do not exist. As a result, they need to divide their time between both parents, increasing the likelihood that they see and support each parent less often (Kalmijn, 2013b).

In addition, this study is also guided by the concept of intergenerational solidarity (Bengtson et al., 2002a; Szydlik, 2012). This concept provides a framework for analyzing both specific behaviors and the emotional bonds shared between family generations in adulthood. It encompasses several key aspects of intergenerational relationships in adulthood, which can be categorized into three dimensions: affectual solidarity, referring to emotional closeness; associational solidarity, involving shared activities and the frequency of contact; and functional solidarity, which includes instrumental support, caregiving, financial assistance, and the sharing of time and space (Szydlik, 2012, 2016).

According to the solidarity concept, parent–child relationships are embedded within the family and, more broadly, within societal and cultural contexts. They are shaped by opportunity structures, such as geographical proximity, family size, and family structure, which can facilitate or hinder interactions between generations. Additionally, they are influenced by the needs of both generations, which may arise from factors such as illness, divorce, or separation. Events like illnesses, for example, can impact an individual family member’s well-being and, in turn, alter family relationships.

## Previous Findings

A key finding from the limited number of longitudinal studies examining the long-term influence of early parent–child relationships is the relatively moderate stability of different emotional components of the parent–child relationship—including emotional closeness, strain, and conflict—from adolescence to adulthood. These studies also indicate that early parent–child relationships have only a small to moderate explanatory power regarding later exchanges and support between generations (Aquilino, 1997; Barnett et al., 2010; Kong & Moorman, 2016; Tubman & Lerner, 1994). According to the research, the early stages of a parent–child relation influence its emotional quality in later life (Tubman & Lerner, 1994) and affect later exchanges of support (Silverstein et al., 2002; Whitbeck et al., 1994). However, while this influence is stronger in the years immediately following adolescence (Thornton et al., 1995), it diminishes over time. As individuals move further from adolescence, the explanatory power of early parent–child relationships decreases, becoming less significant compared to the current life circumstances of both generations (Parra et al., 2015; Tubman & Lerner, 1994).

Furthermore, previous research indicates that while emotional closeness and conflicts in the mother–child relationship during adolescence directly influence emotional closeness in early adulthood, they only indirectly affect the frequency of contact and the provision of support later in life (Rossi & Rossi, 1990; Whitbeck et al., 1994). Additionally, emotional closeness in adulthood enhances the support that adult children provide to their mothers, both directly and indirectly, through the frequency of contact (Cheng et al., 2013; Klaus, 2009). Studies also show that the level of instrumental support adult children offer to their parents is directly influenced by the frequency of intergenerational contact (Swartz, 2009). Geographic distance is another important factor affecting intergenerational solidarity among adults. Shorter distances are associated with more frequent contact and higher levels of personal assistance and support (Hogerbrugge & Komter, 2012; Szydlik, 2012).

One of the most consistent findings regarding the long-term influence of parent–child relationships during childhood and adolescence is the negative impact of parental divorce and separation on the father–child relationship in adulthood (Amato & Sobolewski, 2004; Furstenberg et al., 2015). However, experiencing parental divorce or separation in childhood or adolescence also negatively affects the quality of the adult child’s relationship with their mother, though to a lesser extent. Among children of divorced or separated parents, it is more likely that the relationship with both parents is poor or that the child has a weak relationship with the mother. Research demonstrates that parental divorce or separation in childhood or adolescence has a direct negative effect on the frequency of contact between mothers and children in adulthood and an indirect negative effect on support given to mothers, in particular by sons (Kalmijn, 2013b; Swartz, 2009).

Furthermore, previous research suggests that intergenerational exchange and parent–child relationships in adulthood are influenced by various aspects of family structure, opportunity structure, and the needs of both generations. For instance, studies show that the ages and socioeconomic statuses of both children and parents impact intergenerational relationships in adulthood (Cooney & Dykstra, 2012; Fingerman et al., 2015). Additionally, research indicates that having siblings is associated with greater geographical distance, less frequent contact with parents, and a reduced likelihood of providing instrumental support (Hank & Steinbach, 2015). Moreover, a child’s transition into partnership, marriage, or parenthood not only affects their emotional relationship with their mother (Danielsbacka et al., 2015) but also influences the frequency of contact and the instrumental support they provide (Bucx et al., 2008; Danielsbacka et al., 2015; Hubatkova & Kafkova, 2017; Sarkisian & Gerstel, 2008). Similarly, maternal transitions and life events, such as illness, divorce, separation, and partner loss, have been identified as significant factors shaping mother–adult child relationships and the support adult children provide to their mothers (Fingerman et al., 2011; Kalmijn, 2013a; Tosi & Grundy, 2019).

## Hypothetical Model

**Figure 1.**
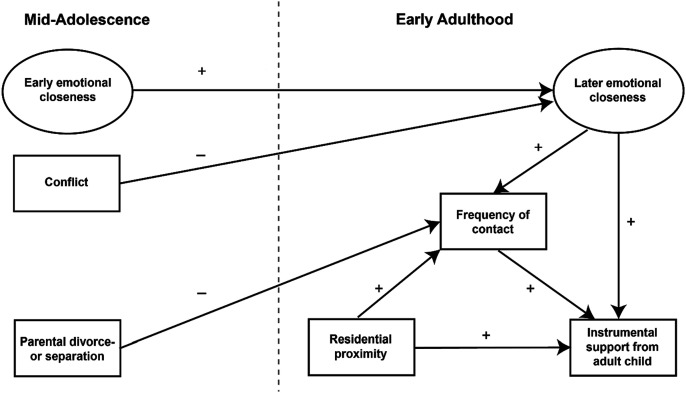
Hypothetical model of the impact of family experiences in mid-adolescence on mother–child relationships in early adulthood.

Based on above considerations, we propose a hypothetical model shown in Figure 1. The model builds on previous research on intergenerational solidarity from a life course perspective (Bengtson et al., 2002a; Rossi & Rossi, 1990; Silverstein & Giarrusso, 2010; Szydlik, 2016). It integrates a longitudinal analysis of stability and change in family relationships from mid-adolescence to early adulthood with both a longitudinal and cross-sectional examination of intergenerational solidarity in early adulthood.

The exogenous variables in the model include emotional closeness in mid-adolescence, conflicts in the parent–child relationship, and parental divorce or separation during childhood or adolescence. Emotional closeness in childhood and adolescence is considered the foundation upon which intergenerational relationships are built later in life. In contrast, conflicts and parental divorce or separation represent potential family stresses and strains during youth, which can have a long-term negative impact on mother–child relationships.

The endogenous variables in the model are emotional closeness and frequency of contact in early adulthood, as well as the instrumental support provided by adult children to their mothers. According to solidarity theory, these aspects of parent–child relationships represent core dimensions of intergenerational relationships in adulthood (Bengtson et al., 2002b; Szydlik, 2016).

In line with previous research (Rossi & Rossi, 1990; Whitbeck et al., 1994), emotional closeness and conflicts in mother–child relationships during mid-adolescence are anticipated to have a direct impact on emotional closeness in early adulthood but only an indirect impact on the later frequency of contact and provision of support. Parental divorce and separation are expected to directly influence the frequency of contact while indirectly impacting the later provision of support to mothers (Kalmijn, 2013b).

In the model, emotional closeness in early adulthood is seen as the dimension of mother–child relationships in adulthood that is most deeply rooted in childhood and adolescence and therefore modeled as having priority over frequency of contact and instrumental support given to mothers in adulthood (Rossi & Rossi, 1990). However, this ordering remains disputed in the literature. Some studies suggest that emotional closeness and frequency of contact in adulthood are mutually dependent upon each other, in accordance with Homans’ (1950) social exchange theory (Silverstein et al., 1995). To take account of this opposing view, a model with a mutual effect of the two dimensions is considered in the empirical analyses as a counter-check.

Moreover, it is assumed that the level of instrumental support provided to mothers in adulthood is directly influenced by the frequency of contact between generations (Swartz, 2009). Furthermore, based on previous research findings, residential proximity is believed to have strong explanatory power for both the frequency of contact and the instrumental support. However, it is not expected to have a direct effect on emotional closeness in adulthood (Hogerbrugge & Komter, 2012; Swartz, 2009). Residential proximity between the two generations plays a special role in the model, as it is considered an important opportunity structure of intergenerational solidarity in adulthood (Bengtson et al., 2002b; Szydlik, 2016).

Finally, as previous research has shown that intergenerational exchange and parent–child relationships in adolescence and adulthood may be influenced by the age and socioeconomic status of both children and parents (Cooney & Dykstra, 2012; Fingerman et al., 2015), we controlled for these variables in the model. Additionally, we accounted for key family structures, opportunity structures, and need structures of both generations (Szydlik, 2016). Specifically, we included the number of siblings, the partnership and parenthood status of adult children, as well as the mother’s illness, the death of her partner, and her divorce or separation in later years.

To test the model and examine differences in mother–daughter and mother–son relationships, structural equation modeling was used.

## Methods

### Sample

Analyses are based on data from the prospective longitudinal LifE study (Fend et al., 2009). The study began in West Germany in the late 1970s and was conducted as a 5-wave longitudinal study of adolescents from 1979 to 1983. A representative sample of 2,030 adolescents, aged 12 to 16, participated in the initial surveys, representing a cohort of late Baby Boomers born between 1963 and 1967 in West Germany (Fend, 1990). Using paper-and-pencil questionnaires, participants reported on family and peer relationships and academic achievement. Data collection in 1982 yielded the most complete data on mother–child relationships in adolescence. After a break of nearly 20 years, the study was resumed in 2002 with the now-adult participants, whose addresses could be found again (*N* = 1853). Participants received a postal questionnaire. Continuing along the same lines as the youth study, the questionnaire focused on family and academic development from adolescence to adulthood and on coping with developmental tasks in early adulthood. A total of 1527 of the former adolescents participated again (completion rate: 82.4%).

The final sample for the analysis of mother–child relationships from mid-adolescence to early adulthood included 1179 participants. Information was available for 612 mother–daughter and 567 mother–son relationships. Data from 1982 to 2002, when participants were 15.56 and 35.42 years old, respectively, were used for this study. Sample statistics are reported in Table 1.

**Table 1.** Sample Statistics: Means, Standard Deviations (SD), and Range.

	Mean	SD	Range
Characteristics of family of origin			
Socioeconomic status at T1 (Kleining & Moore, 1968)	3.94	0.99	1–7
Parents divorced or separated before T1^a^	0.10		0–1
Parents divorced or separated between T1 and T2^a^	0.06		0–1
Father died before T2^a^	0.16		0–1
Characteristics of child			
Female respondent^a^	0.52		0–1
Age at T1	15.56	0.59	14–17
Age at T2	35.42	0.59	34–37
Socioeconomic status at T2 (Wegener, 1988)	53.08	15.57	10–90
Sibling(s) alive at T2^a^	0.88		0–1
Living with spouse/partner at T2^a^	0.79		0–1
Own child/children at T2^a^	0.64		0–1
Characteristics of mother			
Age at T2	61.54	5.21	51–80
Health at T2 (overall physical and mental health)	3.64	0.77	1–5
Mother–child relationship in mid-adolescence (T1)			
Emotional closeness in mother–child relationship	3.47	0.90	1–5
Frequency of conflict in mother–child relationship	2.75	1.06	1–5
Mother–child relationship in early adulthood (T2)			
Emotional closeness in mother–adult child relationship	3.00	0.68	1–4
Frequency of contact in mother–adult child relationship	4.57	1.27	0–6
Frequency of instrumental support from adult child	2.49	1.01	1–5
Geographic distance (km per logarithm)	3.38	1.77	0–7.04

*N* = 1048–1179.

^a^Percentage.

Due to attrition, the study sample in 2002 was slightly underrepresented in terms of adult children with lower socioeconomic status, non-German citizenship, and divorced parents compared to the West German population (Lauterbach et al., 2016). However, the percentages related to geographic distance and the frequency of contact between adult children and parents are comparable to those found in population-based studies of the same cohort in Germany (Hank, 2007).

Geographic distance and intergenerational contact patterns in Germany are similar to those in other Central European countries, such as Austria and France, which have relatively conservative welfare state regimes (Esping-Andersen, 1990; Hank, 2007; Szydlik, 2016). The German welfare system supports traditional family structures and offers a less developed family support system compared to, for example, Scandinavian countries.

### Instruments

All information used to test the hypothetical model is based on children’s reports.

#### Mother–Child Relations in Mid-Adolescence

The measures for mother–child relations are based on the Fend and Prester (1986) instrument on family relationships during adolescence.

*Emotional closeness* with mothers in mid-adolescence was measured as a latent variable consisting of three items (e.g., “My parents/mother always understand/s my problems and support/s me emotionally”; 5-point response scale: “strongly agree” to “strongly disagree”). The measurement models for mothers and daughters, as well as mothers and sons, demonstrate good reliability. They meet the standards of strict factorial measurement invariance (Gregorich, 2006) and have a good global fit (*MLRχ*^*2*^ = 5.749, *p* = .569, *df* = 7, *χ*^*2*^*/df* = 0.821, *CFI* = 1.000, *RMSEA* = 0.000).

The *frequency of conflict* in the mother–child relationship during mid-adolescence was assessed using one item: “My parents/mother and I have a lot of conflicts and quarrels.” Responses were given on a 5-point response scale ranging from “strongly agree” to “strongly disagree.” A limitation of these two measures of mother–child relationships in adolescence is that the wording of the items referred to “parents” rather than exclusively to mothers. However, a control question in the questionnaire revealed that the vast majority of adolescents (94.1%) primarily thought about their mothers, rather than both their mothers and fathers, when answering the questions. Therefore, the two measures can be considered feasible indicators of the quality of mother–child relationships in mid-adolescence.

Information on *parental divorces and separations during childhood and adolescence* (up to the age of 15) was collected from the participants at all points of measurement.

#### Mother–Child Relationships in Early Adulthood

*Emotional closeness in mother–child relations in early adulthood* was measured as a latent variable consisting of four items (with 4-point response scales ranging from “strongly agree” to “strongly disagree”) adapted from the Furman and Buhrmester (1985) instrument on personal relationships and social networks in adulthood. The wording of the items takes into account that the nature of parent–child relationships changes from adolescence to adulthood, evolving into a pattern of increasing symmetry as children grow up. It captures the extent to which the relationship reflects emotional closeness, acceptance, and involvement between grown up children and mothers (e.g., “I love being together with my mother” and “My mother loves me the way I am”). The measurement models for mothers and daughters, as well as mothers and sons, show good reliability. They meet the standards of metric invariance (Gregorich, 2006) and exhibit a good global fit (*MLRχ*^*2*^ = 4.280, *p* = .747, *df* = 7, *χ*^*2*^*/df* = 0.611, *CFI* = 1.000, *RMSEA* = 0.000).

*Frequency of contact with the mother* was assessed with a single item (“How often do you meet your mother?”) on a 7-point response scale (ranging from “daily contact” to “no contact,” with options for “several times a week,” “once a week,” “1 to 3 times a month,” “a few times a year,” and “seldom”).

*Instrumental support for mothers* was measured with a single item that assessed the frequency of help and assistance provided by adult children to their mothers. The item asked, “How often do you help with daily chores, errands, or provide care?” Responses were recorded on a scale from 1 to 5, ranging from “daily” to “never” (with options for “several times a week,” “several times a month,” and “a few times a year”). These items were adapted from the German Aging Survey (Kohli & Kunemund, 2000).

*Residential proximity* between mothers and adult children was measured by one item, as distance in kilometers. To avoid the influence of heteroscedasticity, a log transformation was applied (Hogerbrugge & Komter, 2012).

*Control variables:* The *socioeconomic status* of family of origin (in 1982) and that of adult children (in 2002) was assessed based on information about the job positions of parents and adult children, respectively, coded according to Kleining and Moore (1968) and Wegener (1988). Data on participants’ and mothers’ ages, the presence of siblings, and death of the father, divorce, and separation were collected from the participants at both points of measurement. In 2002, participants reported their partnership status (single, in a partnership, or married) and parenthood status. Additionally, they reported their mothers’ overall physical and mental health using a single item with a 5-point response scale ranging from “very good” to “very bad” health.

## Results

### Mother–Child Relationships in Mid-Adolescence and Early Adulthood

Our analyses confirm the fundamentally positive picture of mother–child relationships reported in the research literature (Steinbach et al., 2020; Treas & Gubernskaya, 2012). The majority of participants in our study felt close to their mothers during mid-adolescence: 85.5% reported that their mothers understood their problems and supported them emotionally (“partially agree,” “agree,” and “strongly agree”). Only 20.2% had frequent conflicts and quarrels with their mothers during mid-adolescence (“agree” or “strongly agree”). 78.5% of the adult children reported that they love being together with their mothers (“agree” or “strongly agree”). The generations mostly live close to one another and maintain frequent contact. 72.4% of adult children live within 25 km (15.5 miles) of their mothers’ homes, and only 1.4% of adult children have moved out of Germany. 81.5% have contact with their mothers at least once a week. 43% of the younger generation provide instrumental support to their mothers at least once a week.

As expected and as reported by previous studies (Cooney & Dykstra, 2012; Swartz, 2009), although both adult daughters and adult sons describe their relationship with their mother as good, there are statistically significant differences between them (Table 2). In general, adult daughters describe their relationship with their mother as closer. They have somewhat stronger emotional ties to their mothers, maintain more frequent contact with them, and provide more help than adult sons. But there is no difference in residential distance in early adulthood between the two mother–child relationships.

**Table 2.** Differences Between Mother–Daughter and Mother–Son Relationships in Early Adulthood.

	Daughters Mean (SD)	Sons Mean (SD)	Differences *p*
Emotional closeness (T2)	3.05 (0.71)	2.95 (0.65)	.003
Frequency of contact (T2)	4.78 (1.22)	4.34 (1.27)	.000
Frequency of instrumental support from adult child (T2)	2.57 (1.05)	2.41 (0.96)	.018
Residential distance (T2, km via logarithm)	3.41 (1.75)	3.35 (1.80)	.709

*Note:* To test for differences between mother–daughter and mother–son relationships, t-tests and non-parametric Mann–Whitney U tests were used.

*N* (daughters) = 597–611. *N* (sons) = 560–565.

### Long-Term Impact of Family Experiences in Mid-Adolescence

To test the proposed model in Figure 1, two alternative multi-group structural equation models were specified and compared: a model with all possible regression paths included and the hypothetical model in Figure 1. Chi-square difference scores (SB*χ*^*2*^_*diff*_) and global fit statistics reveal that the two models are not statistically significantly different from each other. The results of the model comparison are documented in Table 3 (see Models 1 and 2 in Table 3). However, at the item level, the models differ considerably. The proposed hypothetical model proved to be more parsimonious and also more accurate with respect to previous findings in the research literature, with one exception. Contrary to expectations, the empirical analyses detected an additional statistically significant long-term effect of parental divorce and separation in childhood or adolescence on the geographic distance between mothers and adult children. Consequently, a final empirical model, which includes this additional effect, was specified (Model 3 in Table 3). This model has a statistically significantly better global fit than the hypothetical model.

**Table 3.** Comparison of Different Nested Multi-Group Structural Equation Models of the Impact of Family Experiences in Mid-Adolescence on Mother–Child Relationships in Early Adulthood.

Model	MLR*χ*^*2*^	*df*	*χ* ^*2*^ */df*	*SBχ* ^*2*^_*diff*_	CFI	RMSEA
1. Full model with all regression paths specified	335.45***	218	1.54		0.98	0.03
2. Hypothetical model (Figure 1)	378.70***	238	1.59	2.15^n.s.^	0.97	0.03
3. Final empirical model (Figure 2)	361.04***	236	1.53	8.62*	0.98	0.03
4. Non-recursive model	346.15***	232	1.49	3.84^n.s.^	0.98	0.03

*p < .05. **p < .01. ***p < .001.

MLR: Maximum likelihood parameter estimates with standard errors that are robust to non-normality of observations.

MLR*χ*^*2*^*:* Chi-square test statistic that is robust to non-normality of observations.

*SBχ*^*2*^_*diff*_*:* Satorra–Bentler scaled and adjusted chi-square difference test of nested models using MLR under the assumption of non-normality (Satorra, 2000).

CFI and RMESA: Using MLR, fit indices are adjusted for non-normality.

*N* (daughters) = 612. *N* (sons) = 567.

Additionally, to test Homans’ (1950) argument regarding the reciprocal influences between emotional closeness and frequency of contact in mother–daughter or mother–son relationships in early adulthood, a non-recursive model was estimated, incorporating the mutual effects of emotional closeness and frequency of contact in adulthood (Model 4 in Table 3). However, the model could not be confirmed, as no statistically significant reciprocal effects were found.

In all models, missing values were imputed using the Full Information Maximum Likelihood method (FIML). The average rate of missing values per variable in the models was 2.1%. To account for the non-normal distribution of some variables, all analyses were conducted using the Maximum Likelihood Estimation method with Robust Standard Errors (MLR) in Mplus (Muthén & Muthén, 2017). All exogenous variables in the models were allowed to correlate. Bivariate correlations of the model variables are presented in the appendix.

**Figure 2.**
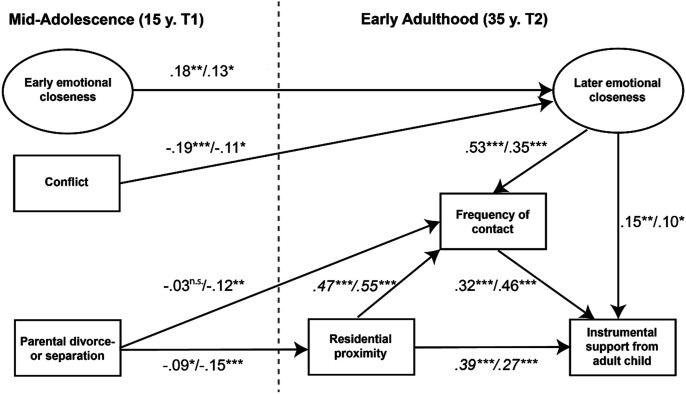
Results of the final empirical model of the impact of family experiences in mid-adolescence on mother–child relationships in early adulthood.

The results of the final empirical model are presented in Figure 2. In the figure, standardized path coefficients are shown. The first path coefficient of a given path reports the results for mother–daughter relationships (*N *= 612), and the second coefficient reports the results for mother–son relationships (*N *= 567). Where there are statistically significant differences between the two relationships, the coefficients are shown in italics.

The results in Figure 2 show a significant association between emotional closeness and conflict in mid-adolescence and emotional closeness in early adulthood for both mother–daughter (emotional closeness β = .18, *p* = .001; conflict β = −.19, *p* = .000) and mother–son relationships (emotional closeness β = .13, *p* = .045; conflict β = −.11, *p* = .048). The effect sizes of the associations are relatively small in absolute terms (Cohen, 1988); however, considering the long observation period of 20 years, they are considerable. The effect sizes for the two mother–child relationships are not statistically significantly different (emotional closeness *p* = .748; conflict *p* = .236). In the full model (Model 1 in Table 3), we tested whether there were any additional direct paths. No other direct effects of these two relationship characteristics in mid-adolescence on frequency of contact, instrumental support given by adult children, or residential proximity between the generations were found. However, further analyses based on the final empirical model (Model 3 in Table 3) revealed that there are statistically significant indirect effects of the two relationship characteristics in mid-adolescence on frequency of contact (mediated by emotional closeness in early adulthood) and instrumental support given by adult children (mediated by emotional closeness and frequency of contact in early adulthood). The parameters of the total indirect effects range from β = │.02│ to β = │.11│. For clarity, these are not included in Figure 2. Detailed information regarding the total indirect effects can be seen in Table 4. Indirect effects in the models were computed using the MLR estimator. To double-check, the bootstrap method was additionally used to estimate bias-corrected standard errors of indirect effects.

**Table 4.** Total Indirect Effects on Frequency of Contact and Instrumental Support Provided to Mothers.

Starting point of indirect effect	Frequency of contact					Instrumental support to mothers				
	Daughters		Sons		Diff.	Daughters		Sons		Diff.
	β	*p*	β	*p*	*p*	β	*p*	β	*p*	*p*
Emotional closeness T1	.11	.001	.05	.046	.319	.04	.003	.02	.047	.605
Frequency of conflict T1	−.11	.000	−.04	.045	.119	−.04	.004	−.02	.050	.298
Parental divorce and separation T1	−.04	.023	−.08	.000	.208	−.01	.402	−.05	.010	.091
Emotional closeness T2						.18	.000	.17	.000	.836
Residential proximity T2						.15	.000	.25	.000	.031

*Note:* Differences in indirect effects were tested by equating the difference terms of both groups using the model constraint option in Mplus.

*N* = 1054–1179.

In summary, results for this part of the model show that emotional closeness in the mother–child relationship in mid-adolescence is directly linked to greater emotional closeness and indirectly linked to more frequent contact and greater support provided by the adult child in later years. Conversely, a relationship with a high level of conflict in mid-adolescence is directly linked to less emotional closeness and indirectly linked to less frequent contact and less support in the later mother–child relationship.

Concerning parental divorce and separation during childhood or adolescence, statistically significant effects on the mother–child relation in adulthood were detected. In the long run, divorce or separation led to greater geographic distance between mothers and children (daughters: β = −.09, *p* = .017; sons: β = −.15, *p* = .000) and a reduction in the frequency of contact between mothers and sons later in life (β = −.12, *p* = .005). Mediated by residential proximity in early adulthood, parental divorce and separation also led to a reduction in the frequency of contact between mothers and adult children (daughters: β = −0.04, *p* = .023; sons: β = −0.08, *p* = .000). Additionally, mediated by residential proximity and frequency of contact, it resulted in a reduction in the instrumental support provided to mothers by adult sons (β = −0.05, *p* = .010; Table 4).

Based on these initial findings, one of the basic assumptions of the postulated model can be confirmed: The results show that family relationship experiences in mid-adolescence, including parental divorce and separation, affect the relationships between mothers and children in early adulthood. Emotional closeness, frequency of contact, and the support provided by children in adulthood can, to a considerable extent, be explained by long-standing characteristics of the relationship that date back to adolescence.

The data also largely confirm additional assumptions concerning intergenerational relationships in adulthood. As Figure 2 shows, emotional closeness in early adulthood not only represents stability in intergenerational relationships but also functions as a strong correlate of the frequency of contact (daughters: β = .53, *p* = .000; sons: β = .35, *p* = .000) and correlates considerably with instrumental support provided by adult children to their mothers (daughters: β = .15, *p* = .003; sons: β = .10, *p* = .019). Emotional closeness in early adulthood can be considered an important prerequisite for contact between mothers and adult children and for instrumental support given to mothers (Cheng et al., 2013; Klaus, 2009). However, in accordance with previous findings (Cooney & Dykstra, 2012; Schwarz, 2006), emotional closeness proved to be independent of residential proximity between generations.

The effects of emotional closeness in early adulthood were not only direct. In addition to direct associations, we found an indirect effect of emotional closeness on instrumental support, mediated by the frequency of contact. This indirect effect has a considerable effect size, is highly significant, and does not differ statistically significantly between daughters and sons (daughters: β = .18, *p* = .000; sons: β = .17, *p* = .000; Table 4).

Residential proximity was found to be a moderate to strong correlate of intergenerational exchange relationships in early adulthood: The opportunity to meet (daughters: β = .47, *p* = .000; sons: β = .55, *p* = .000) and to provide support (daughters: β = .39, *p* = .000; sons: β = .27, *p* = .000) was directly dependent upon geographic proximity. Furthermore, residential proximity also had a substantial indirect effect on instrumental support, mediated by frequency of contact (daughters: β = .15, *p* = .000; sons: β = .25, *p* = .000; Table 4). There was also a moderate to strong direct association between frequency of contact and instrumental support (daughters: β = .32, *p* = .000; sons: β = .46, *p* = .000). It is unsurprising that the closer that mothers and adult children lived to one another, the more contact they had, and the more help mothers received from their adult children. Yet, when it comes to the effect of residential proximity on frequency of contact and support provided, Figure 2 also reveals a difference between mother–daughter and mother–son relationships. Frequency of contact between mothers and sons was found to depend more strongly on residential proximity than was the case with frequency of contact between mothers and daughters (difference in direct effect: *p* = .026). However, in the mother–daughter relationship, there was a stronger association between residential proximity and instrumental support than in the mother–son relationship (difference in direct effect: *p* = .010). There was also a difference between mothers and daughters and mothers and sons with respect to the indirect effect of residential proximity on instrumental support (mediated by frequency of contact). Again, the association was stronger for mothers and sons than for mothers and daughters (difference in indirect effect: *p* = .031; Table 4).

## Discussion

This study aimed to explore the long-term influence of family experiences during mid-adolescence on mother–child relationships in early adulthood and to identify the underlying mechanisms contributing to emotional closeness, frequent contact, and instrumental support provided by adult daughters and sons to their mothers.

Regarding the long-term influence of mother–child relationships in mid-adolescence, the analyses found stability in these relationships for both mothers and daughters, as well as mothers and sons. The relationships between mothers and their adult children appeared, to a considerable extent, to depend on the quality of relationships in the early stages of family life. For example, an emotionally close mother–child relationship in mid-adolescence was directly linked to emotional closeness in early adulthood and indirectly linked to more frequent contact and greater support provided by the adult child.

Furthermore, the findings concerning parental divorce or separation during childhood or adolescence indicate that, in the long run, divorce or separation lead to greater geographic distance between mothers and children. Additionally, it results in a reduced frequency of contact and, indirectly—mediated by the frequency of contact—a decrease in the instrumental support provided by adult sons to their mothers. Thus, our study confirms a well-documented phenomenon in the literature: Compared to children from structurally intact families, adult children of divorced or separated parents—particularly adult sons—distance themselves not only from their fathers but also, albeit to a lesser extent, from their mothers (Kalmijn, 2013b).

These findings must be qualified, in as much as early family experiences in mid-adolescence did not affect the different dimensions of intergenerational relationships in early adulthood to the same extent or in the same way. Whereas emotional closeness in the mother–child relationship in early adulthood was clearly influenced by emotional closeness and the frequency of conflicts in the mother–child relationship in mid-adolescence, the frequency of contact and instrumental support provided to mothers in early adulthood appeared to be more dependent on the opportunity structure of both generations during this phase of life. Emotional closeness in early adulthood emerged as the dimension of intergenerational relationships with the longest history—meaning it is the most affected by early experiences between mothers and children in childhood and adolescence (Rossi & Rossi, 1990). It was also found to be independent of reciprocal influences from the frequency of contact in early adulthood. While the emotional quality of relationships outside the family and relationships with partners and spouses can be strongly influenced by frequency of contact and exchange (Homans, 1950), mother–child relationships in early adulthood are relationships that, in general, build upon a long history of shared experiences and established patterns and thus seem to require less continuous reinforcement through later interactions.

For most participants of this study, the mother–adult child relationship was found to be close and supportive. It appears to constitute a long-lasting emotional bond and function as a latent support network that is activated in times of need (Steinbach et al., 2020; Swartz, 2009).

With regard to mother–daughter and mother–son relationships, this study found that the mother–adult daughter relationship was more strongly characterized by emotional closeness, frequent contact, and support provided than the relationship between mothers and adult sons. In addition, instrumental support provided by adult sons to mothers, compared to support from adult daughters, was found to be somewhat more dependent on the current frequency of contact and possibly also on filial obligations (Silverstein et al., 2006). These findings align with many previous studies conducted in Western societies, which show that women make the greatest contribution to intergenerational relationships (Carr & Utz, 2020; Cooney & Dykstra, 2012). Although we would expect these gender differences given dominant gender roles and socialization, previous analyses suggest that women’s greater involvement in intergenerational relationships also results from structural differences in employment (Sarkisian & Gerstel, 2004).

Some limitations related to the measurement of intergenerational relations from mid-adolescence to early adulthood should be noted. Mother–child relationships in early adulthood were measured cross-sectionally, and therefore, causality between the different dimensions of intergenerational relationships in early adulthood cannot be conclusively ascertained with the present data. More longitudinal data is needed to properly address the question of causality and to clarify the possibility of reverse causation (e.g., if the frequency of contact in early adulthood strengthens emotional closeness or if providing support strains emotional closeness in early adulthood). Furthermore, some measures in the model are feasible but not optimal: The target of the questions used to assess emotional closeness and conflict in the mother–child relationship during mid-adolescence was not restricted exclusively to mothers. Additionally, the frequency of contact and instrumental support provided by adult children in early adulthood, as well as conflict in mid-adolescence, are measures that use single-item indicators.

Despite these limitations, this study replicated many earlier findings and offered new insights into the lasting impact of early family experiences on different dimensions of mother–child solidarity in adulthood. An important strength of this study is the length of the observation period, which spans 20 years, and the availability of longitudinal data on the relationship between two interlinked generations. To our knowledge, no comparable long-term longitudinal data on intergenerational relationships from mid-adolescence to early adulthood of the late Baby Boomer generation in Germany is available.

Altogether, the findings demonstrate that adult children’s emotional closeness with their mothers in early adulthood, as well as their decisions on whether to meet and support them, not only rely on opportunity structures and current parent–child interactions but also on the relationship they had with their mothers while growing up. Some parents may have difficulty establishing warm and affectionate relationships and a new balance of relatedness and autonomy in the relationship with their adolescent offspring. Therefore, social programs that support these parents should be extended. By improving their relationship with their children during adolescence, parents invest in their future intergenerational relationships and the support they will receive from their children in later years.

For future research, it will be important to follow parent–child relationships from childhood and adolescence through mid- and late adulthood, when support from the younger to the older generation becomes even more crucial. Intergenerational relations and caregiving are best understood as lifelong processes, shaped by early family antecedents, short- and long-term impacts, and embedded in cultural and social contexts of development (Szydlik, 2012). They constitute a critical domain in family studies—one that, due to increasing diversity in family forms and growing life expectancy, will likely become even more important in the future (Carr & Utz, 2020).

## Appendix

**Appendix A.** Bivariate Correlations of the Variables in the Models of the Impact of Family Experiences in Mid-Adolescence on Mother–Child Relationships in Early Adulthood.

	1	2	3	4	5	6
1. Emotional closeness T1	_					
2. Frequency of conflict T1	−.44 ***	_				
3. Parental divorce or separation T1	.02 ^ns^	.02 ^ns^	_			
4. Emotional closeness T2	.24 ***	−.24 ***	−.03 ^ns^	_		
5. Frequency of contact T2	.05 ^ns^	−.09 **	−.16 ***	.32 ***	_	
6. Instrumental support to mothers T2	.03 ^ns^	−.08 **	−.12 ***	.23 ***	.66 ***	_
7. Geographic distance T2	.04 ^ns^	.03 ^ns^	.11 ***	−.04 ^ns^	−.61 ***	−.57 ***

*Note:* Spearman’s rho was applied to estimate bivariate correlations.

*N* = 1108–1170. ^ns^ not significant. **p* < .05. ***p* < .01. ****p* < .001.
